# Behavioral and emotional problems in adolescents with constipation and their association with quality of life

**DOI:** 10.1371/journal.pone.0239092

**Published:** 2020-10-12

**Authors:** Shaman Rajindrajith, Nayomi Ranathunga, Nirodha Jayawickrama, Marieke van Dijk, Marc A. Benninga, Niranga Manjuri Devanarayana

**Affiliations:** 1 Faculty of Medicine, Department of Pediatrics, University of Colombo, Colombo, Western Province, Sri Lanka; 2 The Lady Ridgeway Hospital for Children, Colombo, Sri Lanka; 3 Faculty of Medicine, Department of Physiology, Wyamba University of Sri Lanka, Kuliyapitiya, North Central Province, Sri Lanka; 4 National Eye Hospital, Colombo, Western Province, Sri Lanka; 5 Psychological Department, Emma Children Hospital, Amsterdam University Medical Center, Meibergdreef, Amsterdam, The Netherlands; 6 Department of Pediatric Gastroenterology and Nutrition, Emma Children Hospital, Amsterdam University Medical Center, Meibergdreef, AZ, Amsterdam, The Netherlands; 7 Faculty of Medicine, Department of Physiology, University of Kelaniya, Ragama, Western Province, Sri Lanka; Temple University, UNITED STATES

## Abstract

**Objectives:**

To assess behavioral and emotional problems in children and adolescents with functional constipation and their relationship with psychological maladjustment and health-related quality of life (HRQoL).

**Design:**

A school-based cross-sectional survey conducted in 8 randomly selected schools from 4 randomly selected districts in Sri Lanka. A previously validated questionnaire was used for data collection. Behavioral and emotional problems were assessed using the Sinhala version of the Child Behavior Check List (CBCL-S/4-18). Constipation was diagnosed by applying the Rome III criteria.

**Results:**

A total of 1000 questionnaires were distributed, and 913 completed questionnaires were included in the analysis. Sixty adolescents (6.5%) had functional constipation. Scores obtained for isolated psychological problems such as withdrawal (3.1 [3.1] vs. 1.9 [2.4], *p*<0.001), somatic complaints (3.2 [2.8] vs. 2.3 [2.5], *p*<0.05) anxiety/depression (5.8 [2.5] vs. 3.9 [3.6], *p*<0.001), social problems (3.0 [2.7] vs. 2.2 [1.9] *p*<0.001) and attention problems (5.4 [4.1] vs. 3.9 [3.4], p<0.001), and broadband scale of internalization (12.1 [8.4] vs. 8.3 [7.2], *p*<0.05) and mean total CBCL-S/4-18 score (29.4 [19.5] vs. 23.2 [17.0], *p*<0.001) were higher in adolescents with functional constipation. Clinical characteristics, socio-demographic and family factors and psychological maladjustment had no relationship with externalization, internalization and total CBCL-S/4-18 score. Internalization (-0.49, *p*<0.0001), externalization (-0.30, *p*<0.05), and total CBCL-S/4-18 (-0.44, *p*<0.001) scores had a negative impact on HRQoL of adolescents with functional constipation.

**Conclusions:**

Adolescents with functional constipation are suffering from significant behavioral and emotional problems. These problems negatively affect their HRQoL.

## Introduction

Functional constipation (FC) in children and adolescents is a common problem with a worldwide prevalence of around 9.5% [1]. It negatively affects all areas of health-related quality of life (HRQoL) [2]. FC also has a significant impact on the healthcare system due to increasing numbers of such patients and escalating healthcare costs [3,4]. Families with affected adolescents often face social isolation and financial constraints [5]. Besides, they suffer from a multitude of extra-intestinal somatic symptoms that amplify their suffering [6,7].

Although the etiology of FC is mostly unknown, there had been an accumulation of data that points out the presence of psychological and behavioral problems in children of all ages with FC. A recent study in Sri Lanka reported psychological maladjustment in adolescents with constipation [8]. In contrast, younger children who were suffering from constipation were noted to have more anxiety and depression [9,10], emotional problems, and problems with peers [10]. Children investigated and treated in hospitals have more severe symptoms and do not represent other children with less severe symptoms. Although adolescents were included in some of these studies, their number is low, and these studies have not focused on assessing the behavioral and emotional problems in adolescents specifically. Therefore, we set out to gain an understanding of the behavioral and emotional problems of adolescents with FC in the community. We also wanted to know how these problems affect symptoms, psychological maladjustment, and health-related quality of life (HRQoL).

## Methods

### Sample

We conducted a cross-sectional survey in 4 randomly selected districts (out of 25) of Sri Lanka. Two schools (with both girls and boys) were randomly selected from each district (a total of 8) using the list of schools available in the Zonal Education Director's Offices in the respective districts. From each school, grades 9 to 13 were included in the study. All adolescents aged between 13 and 18 years in the selected classes of the schools were invited to participate in the study.

### Data collection

The study was carried out from January 2014 to January 2015. We visited the selected schools 2 days before data collection and handed over the information sheet and consent forms to the students. A full explanation regarding the study was provided for all prospective participants. We requested them to take the documents home and get consent from parents to participate in the study. We included all adolescents who had a written affirmation from their parents as well as personal assent from each student.

The research assistants supervised data collection. Adolescents completed the questionnaire for functional gastrointestinal disorders as well as the one for personality assessment and quality of life in the school. They were given the CBCL-S/4-18 and the information sheet related to the questionnaire and requested to hand it over to their parents. The parents were invited to complete the CBCL-S/4-18 and return them to the class teacher. Research assistants collected the questionnaires from the class teachers. Two reminders were sent to parents who failed to return the questionnaire to optimize the response rate.

The following questionnaires were used in the data collection. All these tools have been previously translated and validated and used in studies in Sri Lankan children and adolescents.

1. Questionnaire on childhood functional gastrointestinal disorders
  This questionnaire was based on the Rome III Diagnostic Questionnaire for Pediatric Functional Gastrointestinal Disorders [11]. It has been previously used in Sri Lankan studies [8,12–15].
2. Child Behavior Checklist (CBCL)
  The CBCL is a caregiver report that helps to identify behavioral and emotional problems in children. The CBCL school-age version (CBCL/4-18) uses the data provided by a respondent who knows the child well (usually a parent). The checklist has 118 questions that help to identify 8 empirically-based clinical syndromes. They include anxiety/depression, somatic complaints, withdrawal, attention problems, social problems, thought problems, delinquency, and aggressive behavior. Using some of them collectively, it is possible to identify 2 broadband behavioral scales, namely internalization (anxiety/depression, somatic complaints, withdrawal) and externalization (delinquency and aggressive behavior). The total score is computed by summing up the scores for all 8 syndromes [16]. The native Sri Lankan language Sinhala version of the questionnaire (CBCL-S/4-18) has been translated and validated using standard methods in the past [17]. The Cronbach alpha for the internal consistency of the CBCL-S/4-18 was reported as 0.74 (internalization 0.69, externalization 0.79) for boys and 0.73 (Internalization 0.69, externalization 0.79) for girls [17].
3. Childhood personality assessment questionnaire (Child PAQ)
  Child PAQ is a self-administered instrument to assess psychological maladjustment. It assesses an individual's perception of himself or herself concerning seven personality dispositions (hostility and aggression, dependency, negative self-esteem, negative self-adequacy, emotional unresponsiveness, emotional instability, and negative world view) [18].
  The Child PAQ has six items for each of the seven personality dispositions. The child PAQ asks respondents to reflect on their actual, feelings about themselves. The response options available are: (1) almost always true of me; (2) sometimes true of me; (3) rarely true of me; and (4) almost never true of me. Summing up the scores of each of the seven scales, an overall assessment of the level of personality development of the respondent was made. Higher total scores reflect imperfect personality development. It was translated and validated with acceptable rates for the content and consensual related validities and a significant Kappa value of agreement (0.82) between clinician's rating and the PAQ [19,20].
4. Pediatric Health-related quality of life inventory (PedsQL)
  PedsQL is a tool used to assess HRQoL in adolescents. We used the Quality of Life Inventory for teenagers 13–18 years of age [21]. It is a self- reported questionnaire that has been pretested for the relevant age group. It has previously undergone linguistic validation, pretested for Sri Lankan adolescents of this age group, and used in several Sri Lankan studies [7,22,23]. The internal consistency has not calculated for the local language version of this questionnaire. However, in previous studies, Cronbach’s alpha for internal consistency for the full scale is reported to be 0.88 for English version [21].

The inventory has 23 items and encompasses physical (8 items), emotional (5 items), social (5 items), and school functioning (5 items). A 5-point response scale is used (0 = never a problem; 4 = almost always a problem). Items were reverse-scored and linearly transformed to a zero to 100 scale (0 = 100, 1 = 75, 2 = 50, 3 = 25, 4 = 0) with higher scores indicating better health-related quality of life. Final HRQoL scores were computed out of 100.

### Diagnostic criteria for functional constipation

Functional constipation in adolescents was diagnosed using the Rome III criteria [24]. Stool consistency was determined using the Bristol Stool Scale form [25].

The diagnosis of functional constipation must include two or more of the following in a child with a developmental age of at least 4 years with insufficient criteria for the diagnosis of IBS;

1. Two or fewer defecations in the toilet per week
2. At least 1 episode of fecal incontinence per week
3. History of retentive posturing or excessive volitional stool retention
4. History of painful or hard bowel movements
5. Presence of a large fecal mass in the rectum
6. History of large diameter stools that may obstruct the toilet
  These criteria fulfilled at least once per week for at least 2 months before diagnosis.

### Statistical analysis

Data from all 8 schools were pooled for the initial analysis. PSPP version 0.8.3-g5f9212 statistic software (Free Software Foundation, Inc.http://fsf.org/) was used in data analysis. Chi-Square test was used to analyze the association between constipation and CBCL-S/4-18 using this software. Since the T-score norms of the CBCL/4-18 are not available for adolescents in Sri Lanka, we used the raw scores. Means and standard deviations were calculated for data obtained for CBCL/4-18, HRQoL, and PAQ, were compared using unpaired t-test. '*p'* values were two-sided, and the minimum statistically significant level was defined as p<0.05.

### Ethical approval

Ethics Review Committee of the Sri Lanka College of Pediatricians approved the study protocol (Reference No. SLCP/ERC/2011/04).

## Results

### Sample characteristics

A total of 1000 questionnaires were distributed and properly completed 913 (91.3%) were included in the analysis. Sixty (60) adolescents fulfilled the Rome III criteria for FC (mean age 14.3 [SD 1.6] years), giving a prevalence of 6.5% of which only two adolescents had fecal incontinence at least once a week. Adolescents with FC were compared with 853 adolescents without FC (according to Rome III criteria) who were considered as controls (mean age 14.5 [SD 1.8] years). **Table 1** shows the socio-economic and family characteristic of the study sample. There was no significant difference between the two groups.

**Table 1:** Socio-demographic and family characteristics of the study sample.

Characteristics		Constipation	Controls
		(*n* = 60)	(*n* = 853)
		*n* (%)	*n* (%)
Number of children in family	1	07 (11.7%)	73 (8.6%)
	2	23 (38.3%)	313 (36.7%)
	3	12 (20.0%)	245 (28.7%)
	4 or more	18 (30.0%)	222 (26.0%)
Birth order	1	22 (36.7%)	417 (48.9%)
	2	26 (43.3%)	290 (34.0%)
	3	10 (16.7%)	96 (11.3%)
	4 or more	02 (3.3%)	47 (5.5%)
	No response	00	3 (0.3%)
Monthly family income	<10000	12 (20.0%)	173 (20.3%)
(Sri Lankan Rupees)	10000–25000	33 (55.0%)	401 (47.0%)
	>25000–50000	11 (18.3%)	198 (23.2%)
	>50000–100000	04 (6.7%)	43 (5.0%)
	>100000	00	07 (0.8%)
	No response	00	31 (3.6%)
Father’s occupation	Higher profession	03 (5.0%)	25 (2.9%)
	Lesser profession	06 (10.0%)	88 (10.3%)
	Skilled non-manual	18 (30.0%)	242 (28.4%)
	Skilled manual	23 (38.3%)	344 (40.3%)
	Unskilled/unemployed	10 (16.7%)	152 (17.8%)
	Dead	0	02 (0.2%)
Mother's occupation	Higher profession	03 (5.0%)	07 (0.8%)
	Lesser profession	06 (10.0%)	85 (10.0%)
	Skilled non-manual	05 (8.3%)	47 (5.5%)
	Skilled manual	03 (5.0%)	44 (5.2%)
	Unskilled/unemployed	41 (68.3%)	669 (78.4%)
	Dead	02 (3.3%)	01 (0.1%)
Living area	Galle	18 (30.0%)	247 (29.0%)
	Gampaha	21 (35.0%)	208 (24.4%)
	Matara	10 (16.7%)	170 (19.9%)
	Rathnapura	11 (18.3%)	228 (26.4%)

*p* value >0.05 for all comparisons between constipation and controls, chi-square test.

### Association between behavioral problems and FC

Scores for behavioral problems are depicted in **Table 2**. According to the data, scores obtained for withdrawal (3.1 [3.1] vs. 1.9 [2.4], p<0.001), somatic complaints (3.2 [2.8] vs. 2.3 [2.5], p<0.05) anxiety/depression (5.8 [2.5] vs. 3.9 [3.6], p<0.001), social problems (3.0 [2.7] vs. 2.2 [1.9] p<0.001) and attention problems (5.4 [4.1] vs. 3.9 [3.4], p<0.001) were significantly higher among adolescents with FC. Internalization score (12.1 [8.4] vs. 8.3 [7.2], p<0.05), and mean total CBCL-S/4-18 score (29.4 [19.5] vs. 23.2 [17.0], p<0.001) were also significantly higher in adolescents with FC.

**Table 2:** Scores obtained for CBCL-S/4-18 in adolescents with FC and controls.

Clinical syndrome	Constipation	Controls	*Effect size*	*p* value*
	(*n* = 60)	(*n* = 853)	Cohen’s d	
	*Mean (SD)*	*Mean (SD)*		
Withdrawn	3.1 (3.1)	1.9 (2.4)	0.43	<0.0001
Somatic complaints	3.2 (2.8)	2.3 (2.5)	0.34	0.01
Anxious/depressed	5.8 (4.5)	3.9 (3.6)	0.47	<0.0001
Social problems	3.0 (2.7)	2.2 (1.9)	0.34	0.003
Thought problems	1.2 (1.4)	0.9 (1.4)	0.21	0.23
Attention problems	5.4 (4.1)	3.9 (3.4)	0.40	0.002
Delinquent behavior	0.9 (1.3)	0.9 (1.3)	0.0	0.79
Aggressive behavior	7.1 (5.7)	6.2 (5.2)	0.16	0.20
Internalization	12.1 (8.4)	8.3 (7.2)	0.49	<0.0001
Externalization	8.3 (6.6)	7.0 (6.0)	0.21	0.15
Total CBCL-S/4-18 score	29.4 (19.5)	23.3 (17.0)	0.33	0.01

* Unpaired t test.

### Clinical characteristics and CBCL-S/4-18 in adolescents with FC

**Table 3** shows the internalization, externalization, and total CBCL-S/4-18 scores obtained by adolescents with FC, according to clinical characteristics. Two broadband scales and the total CBCL-S/4-18 score show no difference according to clinical characteristics.

**Table 3:** Scores obtained for CBCL-S/4-18 according to clinical characteristics in adolescents with FC.

		Total CBCL score		Internalization score		Externalization score	
		Mean (SD)	*p* value*	Mean (SD)	*p* value*	Mean (SD)	*p* value*
Stool frequency <3 times per week	Yes (*n* = 18)	29.8 (16.7)	0.91	13.0 (8.2)	0.58	7.2 (4.6)	0.41
	No (*n* = 36)	29.1 (20.9)		11.7 (8.5)		8.7 (7.4)	
Hard tool	Yes (*n* = 11)	34.6 (31.2)	0.35	12.8 (10.0)	0.76	10.6 (11.4)	0.22
	No (*n* = 49)	28.2 (16.0)		12.0 (8,1)		7.7 (5.1)	
Pain during defecation	Yes (*n* = 35)	28.4 (15.8)	0.68	11.8 (8.4)	0.75	8.3 (5.0)	0.91
	No (*n* = 25)	30.7 (23.9)		12.6 (8.5)		8.1 (8.6)	
Large volume stool	Yes (*n* = 35)	26.5 (16.0)	0.19	10.7 (6.4)	0.10	7.4 (5.0)	0.23
	No (*n* = 25)	33.8 (23.7)		14.3 (10.5)		9.6 (8.6)	
Stool withholding behavior	Yes (*n* = 32)	29.3 (23.5)	0.97	11.6 (8.6)	0.62	7.9 (7.9)	0.67
	No (*n*-28)	29.5 (13.3)		12.7 (8.2)		8.7 (4.9)	
Fecal incontinence	Yes (*n* = 2)	11.0 (4.2)	0.18	5.0 (1.4)	0.22	3.0 (1.4)	0.26
	No (*n* = 58)	30.1 (19.5)		12.4 (8.4)		8.4 (6.7)	
Urgency	Yes (*n* = 23)	28.9 (17.0)	0.88	11.5 (7.8)	0.64	7.8 (5.1)	0.66
	No (*n* = 37)	29.7 (21.2)		12.5 (8.8)		8.6 (7.6)	
Straining	Yes (*n* = 21)	28.3 (26.9)	0.75	11.4 (9.2)	0.62	7.9 (9.2)	0.77
	No (*n* = 39)	30.0 (13.8)		12.5 (8.0)		8.5 (4.6)	
A feeling of incomplete evacuation	Yes (*n* = 21)	32.7 (16.8)	0.35	12.7 (7.3)	0.71	9.0 (4.9)	0.53
	No (*n* = 39)	27.5 (20.7)		11.8 (9.0)		7.8 (7.5)	
Abdominal pain	Yes (*n* = 28)	28.7 (15.9)	0.82	11.0 (7.2)	0.31	8.1 (5.0)	0.91
	No (*n* = 32)	29.9 (22.6)		13.2 (9.3)		8.3 (8.0)	
Nausea	Yes (*n* = 16)	38.7 (13.1)	0.22	17.3 (7.7)	0.11	9.7 (3.10	0.59
	No (*n* = 44)	28.2 (19.9)		11.5 (8.3)		8.1 (7.0)	
Bloating	Yes (*n* = 20)	35.0 (24.8)	0.12	13.5 (9.8)	0.37	9.1 (8.9)	0.52
	No (*n* = 40)	26.3 (15.4)		11.4 (7.6)		7.8 (5.2)	

* Unpaired t-test.

### Correlation between CBCL-S/4-18 scores and psychological maladjustment in adolescents with FC

**Table 4** shows the scores obtained for psychological maladjustment and personality dispositions in adolescents with FC and controls. Scores obtained for dependency, emotional unresponsiveness, emotional instability and total personality score were higher in adolescents with FC.

**Table 4:** Scores obtained for personality dispositions in adolescents with FC and controls.

	Constipation	Controls	*Effect size*	*p* value*
	(*n* = 60)	(*n* = 853)	Cohen’s d	
	Mean (SD)	Mean (SD)		
Hostility and aggression	14.6 (4.2)	14.1 (4.7)	0.11	0.42
Dependency	16.1 (6.7)	14.0 (7.0)	0.31	0.02
Negative self-esteem	14.4 (2.8)	14.5 (2.5)	0.04	0.80
Negative self-adequacy	14.7 (3.0)	14.2 (3.1)	0.16	0.17
Emotional unresponsiveness	15.5 (3.7)	14.0 (3.7)	0.41	0.001
Emotional instability	15.3 (3.6)	14.1 (3.8)	0.32	0.01
Negative world view	13.7 (2.8)	14.2 (3.1)	0.17	0.24
Total personality score	104.3 (18.0)	99.0 (18.7)	0.29	0.03

* Unpaired t test.

**Table 5** shows the correlation between total CBCL-S/4-18 score and psychological maladjustment. We found no influence of internalization, externalization and total CBCL-S/4-18 score on the seven personality dispositions and psychological maladjustment.

**Table 5:** Correlation between scores obtained for CBCL-S/4-18 and personality types in adolescents with FC.

	Total CBCL-S/4-18 score	Internalization	Externalization
Personality type	*Correlation coefficient*	*Correlation coefficient*	*Correlation coefficient*
Hostility and aggression	0.03	0.08	0.03
Dependency	0.01	-0.03	0.05
Negative self-esteem	0.02	0.01	0.03
Negative self-adequacy	-0.05	-0.05	0.02
Emotional unresponsiveness	0.03	0.03	0.04
Emotional instability	-0.01	-0.1	0.02
Negative world view	0.03	0.04	0.02
Total personality score	0.01	0.01	0.04

*p*>0.05 for all correlations, Pearson correlation coefficient.

### Health-related quality of life and CBCL-S/4-18 score in adolescents with FC

**Table 6** tabulates the details of HRQoL in adolescents with FC. According to the data, scores obtained for internalization and externalization and the total CBCL-S/4-18 score have significant negative correlations with several domains of the HRQoL and the total HRQoL score.

**Table 6:** Correlation between scores of the CBCL-S/4-18 and quality of life in adolescents with FC.

*Quality of life domains*	Total CBCL-S/4-18 score	Internalization	Externalization
	*Correlation coefficient*	*Correlation coefficient*	*Correlation coefficient*
Physical functioning	-0.24*	-0.27*	-0.16*
Emotional functioning	-0.26*	-0.32*	-0.20*
Social functioning	-0.23*	-0.25*	-0.14*
School functioning	-0.25*	-0.26*	-0.21*
Total QOL score	-0.31*	-0.34*	-0.22*

*p< 0.001, Pearson Correlation Coefficient.

## Discussion

In this population-based study, we evaluated the behavioral and emotional problems of adolescents with FC and their correlation with psychological maladjustment and HRQoL. We found that adolescents with FC are at a higher risk of having significant behavioral and emotional or internalizing problems, including withdrawal, somatic complaints, anxiety, and depression compared to their healthy counterparts. Furthermore, they also tend to have social and attention problems and internalization as well. The CBCL-S/4-18 broadband scales (internalization, externalization) and the total score showed a negative impact on HRQoL in adolescents with FC.

The findings of the current study are partly supported by two hospital-based studies on adolescents with FC [9,26]. A study from Turkey reported higher rates of withdrawal, anxiety, depression, somatization, and aggression in adolescents with FC [9]. In addition, van Dijk et al., reported a higher number of children and adolescents with FC (mean age 6.7 years) with overall, internalizing and externalizing behavior in adolescents with FC (mean age of 6.7 years) attending a tertiary care gastroenterology clinic [26]. In contrast to the findings of both these studies [9,26], we did not find higher scores for externalization in our adolescents with FC. Both these studies have included adolescents from tertiary care hospitals who have more severe symptoms. We speculate that more severe behavioral problems seen in these two studies could be attributed to the severity of symptoms.

In this study, we failed to find an association between clinical symptoms of FC and externalization and internalization and the total CBCL-S/4-18 scores. Similarly, Klinicaslan et al. did not report an association between symptoms and components of CBCL [9]. A Dutch study, however, reported an association between externalization and frequency of fecal incontinence and large diameter stools [26]. Dutch children had a higher frequency of fecal incontinence (28.6% at least 1–3 times per week), indicating the severity of constipation [26], compared to our data (only two adolescents had fecal incontinence once a week). Data from the Turkish study, of whom 12% of the children with FC had fecal incontinence more than 2 per week [9]. This could have contributed to the lack of association between broadband scales and total CBCL-S/4-18 scores with clinical characteristics in our study.

Psychological comorbidities are common in children suffering from chronic diseases [27,28]. In this study, we found that dependence, emotional unresponsiveness, and emotional instability were more common among children with FC, similar to that reported previously [8]. We also concentrated on the correlation between broadband scales and the total scores of the CBCL-S/4-18, and personality dispositions in the PAQ. None of the components of the CBCL-S/4-18 showed a significant correlation with the 7 personality dispositions that contributed to psychological maladjustment.

In addition, we reported the correlation between behavioral problems and HRQoL in adolescents with FC. We found that the broadband scale of internalization negatively affects all domains of HRQoL, whereas externalization only had a negative impact on physical functioning and social functioning. Total CBCL-S/4-18 score had negative correlations with all domains and a total score obtained for HRQoL. This finding is consistent with a recent paper, where we reported that psychological maladjustment also negatively affects HRQoL in adolescents with FC [8]. Both symptoms of constipation and the effects of psycho-pathological functioning that contribute to broadband scales could have contributed to the lower HRQoL in these adolescents.

There are several strengths to this study. We included a large number of adolescents in the study to give us sufficient power to interpret the data. This also included a much larger control population in comparison with the cases. All the questionnaires included in the study were previously validated in Sri Lanka using standard techniques. However, we also acknowledge several drawbacks of our study. In this study, the diagnosis of FC was only based on a questionnaire. As in any community-based study, we were also unable to conduct a complete physical examination or investigations to rule out organic disorders. However, organic disorders presenting as constipation are rare in children [29]. Although we found several behavioral and emotional correlates with FC, it is not possible to determine a causal association between them and FC. Finally, it is difficult to determine whether FC predisposed adolescents to develop behavioral problems or vice versa.

We want to highlight that a significant proportion of adolescents with FC are suffering from behavioral and emotional problems that can easily be identified using the CBCL-S/4-18. Behavioral and emotional problems have a negative impact on their HRQoL. Therefore, we believe that screening for behavioral and emotional problems would be a valuable part of the assessment of adolescents with FC. The help of a psychologist needs to be sought when the scores are on or close to the clinical range to ensure optimal care of these adolescents.

## Supporting information

S1 File (XLSX)
